# Pre-cueing, Perceptual Learning and Cognitive Penetration

**DOI:** 10.3389/fpsyg.2017.00739

**Published:** 2017-05-10

**Authors:** Dimitria Electra Gatzia, Berit Brogaard

**Affiliations:** ^1^Department of Philosophy, University of Akron Wayne CollegeAkron, OH, USA; ^2^Centre for Philosophical Psychology, University of AntwerpAntwerp, Belgium; ^3^Philosophy, University of MiamiMiami, FL, USA; ^4^Department of Philosophy, University of OsloOslo, Norway

**Keywords:** cognitive penetration of perception, covert endogenous attention, perceptual learning, visual perception, cognition

In *The Principles of Psychology*, James (1981) suggested that attending to a stimulus can make it appear more “vivid and clear.” Pre-cueing, the procedure in which a cue stimulus is presented to direct a subject's attention to the location of a test stimulus, has been used to test James' hypothesis (Posner, 1978, 1980; Carrasco et al., 2004, 2006; Yeshurun and Rashal, 2010; Carrasco, 2011; Kravitz and Behrmann, 2011). A recent debate concerns whether pre-cueing effects associated with covert attention involve cognitive penetration. In the context of information processing, cognitive penetration occurs when the information content of cognitive states directly influences perceptual computations in such a way as to alter their output^1^.

Attention is the process that either enhances the representation of relevant information (e.g., a scene at a certain location or an aspect of a visual scene) at the system level or diminishes the representation of irrelevant noise. Studies show that attention boosts the apparent stimulus contrast (Carrasco et al., 2004) and increases contrast sensitivity, which seems to be mediated by contrast grain—an effect akin to a change in the physical contrast stimulus (Carrasco et al., 2006). In addition, attention enhances spatial identification accuracy (Yeshurun and Rashal, 2010) and aids in the segmentation of the retinal image by increasing both first- and second-order sensitivity to the attended location (Barbot et al., 2012).

Attention can be allocated either by some bodily orientation of the organs by which an organism moves, say, when the eyes move in the direction of a target location (overt attention) or by shifting the direction of attention without reorienting the body, say, when attention is drawn by the salience of a cue while the eyes remain fixed (covert attention, see Findlay and Gilchrist, 2003). Cases that involve overt attention are not treated as cases of cognitive penetration because overt attention functions as a passive partition mechanism acting prior to the beginning of perceptual process (Pylyshyn, 1999; Macpherson, 2012; Deroy, 2013; Mole, 2015; Firestone and Scholl, 2016; Brogaard and Gatzia, 2017). Recently, however, it has been suggested that cases of covert attention are instances of cognitive penetration (Mole, 2015; Wu, in press) because covert attention functions as an active controlling influence of perceptual processing (Nanay, 2010; Carrasco, 2011, 2014).

Overt and covert attention can be exogenous or endogenous. Exogenous attention corresponds to a reflexive, involuntary response to a location upon the occurrence of a sudden or intense stimulation—it is oriented in a stimulus-driven or bottom-up manner (Carrasco, 2006, 2011). For example, when letters appear abruptly on a computer screen, they capture the eyes' attention and elicit faster responses than when they appear gradually (Yantis and Jonides, 1984; Jonides and Yantis, 1988). Endogenous attention corresponds to our ability to monitor stimulus information (typically based on a cue) voluntarily—it is oriented in a goal-driven or top-down manner (Carrasco, 2006, 2011). For example, we often prepare for an expected event by orienting attention to a location or an object and to the time of the event (LaBerge, 1995). Covert endogenous attention is, therefore, the most relevant type in the debate about cognitive penetration.

Wu (in press) defines attention as *selection for action* (see also Wu, 2014) and argues that covert (endogenous) attention penetrates visual processing. The claim that action requires attention is supposed to follow from what Wu (2011) calls the “Many-Many Problem,” which can be described as follows. A subject is confronted with multiple targets, say, a football or a soccer ball simultaneously, and has the option of either kicking the soccer ball with the right foot or the football with the left foot. While keeping her gaze fixed, she kicks the soccer ball with the right foot, even though she could have kicked the football with the left foot. The supposition that the subject's gaze remains fixed is needed to ensure that this is a case of covert attention (and not a case of overt attention, where cognition controls visual experience by controlling what gets into the mind via the eyes). According to Wu, a consequence of the selection for action view is that when a subject selects a target to guide response in a task (e.g., kick the soccer ball) that subject attends to a target.

It has been suggested that Wu's response to the Many-Many Problem is based on an oversimplified distinction between actions and reflexes (see Jennings and Nanay, 2016). Wu distinguishes between actions, which require a many-many mapping between stimulus and response (e.g., kicking the soccer ball with the right foot or kicking the football with the left foot) and pure reflexes, which exhibit only a one-one mapping between stimulus and response (Wu, 2011, p. 54). However, this “one-one mapping” claim can take either a weak or a strong modal reading: on the weak reading, each type of stimulus corresponds to one type of response; on the strong reading, the stimulus type necessitates a type of response (see Jennings and Nanay, 2016). Both readings are problematic. The weak reading is too weak to distinguish pure reflex from action (for example, ordering the same type of coffee every time you visit your favorite cafe would be a case of reflex in this reading). The strong reading is too strong to capture paradigmatic cases of pure reflex (for example, cases of the knee-jerk reflex where the stimulus is present without the triggering kick response) (Jennings and Nanay, 2016).

Leaving this issue aside, let us return to the question of whether covert endogenous attention penetrates perceptual processes. Clearly, not all cases of covert attention involve cognitive penetration. The consensus is that cases of ambiguous figures are not instances of cognitive penetration because what triggers the changes in the perceptual process is that “different image regions are selectively processed over others because such regions are attended differently in relatively peripheral ways” (Firestone and Scholl, 2016, p. 14; see also Raftopoulos, 2013). There, however, additional reasons for denying that covert endogenous attention penetrates perceptual processes. Specifically, it seems that pre-cueing effects associated with covert attention are similar to perceptual learning effects, despite the former having more transient effects on attention than the latter.

Perceptual learning involves relatively long-lasting (developmental or evolutionary) changes to an organism's neural circuitry associated with specific ecological factors, which typically improve its ability to respond to its environment (see Gibson and Gibson, 1955; Hall, 1991; Karni and Sagi, 1991; Fahle and Morgan, 1996; Bruce and Burton, 2002; Purves et al., 2015). Attentional weighting^2^ involves relatively long-lasting changes to an organism's perceptual system that allow it to increase attention allocated to stimuli that have significance and decrease attention allocated to extraneous stimuli (Goldstone, 1998).

Covert endogenous attention modulates visual processes that occur early on in the processing hierarchy (as does covert exogenous, albeit via different mechanisms; for reviews see Carrasco, 2011 and Pinto et al., 2013)^3^. For example, covert endogenous attention has been found to boost luminance contrast (first-order information) and contrast sensitivity (second-order information; Carrasco et al., 2004; Barbot et al., 2012), enhance spatial resolution (Carrasco et al., 2006), and reduce crowding (Yeshurun and Rashal, 2010). However, the current evidence indicates that there are clear constraints on top-down influences at all levels of information processing (Knill and Richards, 1996; for a review see Teufel and Nanay, 2017).

Typically, high-level cognitive processing influences low-level perceptual processing by facilitating the integration of relevant information at a global level with sensory inputs (Brunner and Goodman, 1947; Knill and Richards, 1996). The ultimate aim of top-down influences is the optimization of information processing at the system level, which is largely attributable to the fact that organisms exploit the statistical properties of natural scenes (Simoncelli and Olshausen, 2001; Geisler, 2008; Purves et al., 2015). For example, when retinal image information is inadequate, the perceptual system relies more heavily on prior probability distributions of different possible environmental states and adjusts its estimates accordingly (Knill and Richards, 1996; Torralba, 2003).

Covert endogenous attention can adjust to optimize performance depending on task demands (Barbot et al., 2012; see also Lee and Schmidt, 2005). This flexibility, however, comes at a cost. Attentional highlighting of information occurs even when it has adverse behavioral effects. For example, a letter which was used consistently as the target in a detection task becomes a distractor (i.e., the stimulus to be ignored), it automatically captures attention (Shiffrin and Schneider, 1977). The converse effect, i.e., negative priming, also occurs. Subjects respond to targets that served as distractors in the past more slowly than newly introduced stimuli (Tipper, 1992) while the effect of such previous exposures can last upward of 2 weeks (Fox, 1995). These studies show that pre-cueing effects associated with covert attention are similar to perceptual learning effects associated with attentional weighting. The main difference is that the former have more transient effects than the latter. While some consider perceptual learning to be a special case of cognitive penetration, viz. diachronic cognitive penetration (Churchland, 1988; Cecchi, 2014), the general consensus is that perceptual learning does not involve cognitive penetration (Fodor, 1988; Raftopoulos, 2001; Arstila, 2016).

Thus far, we pre-supposed that covert endogenous attention differs significantly from covert endogenous attention. However, studies indicate that the oculomotor system (the part of the central nervous system having to do with eye movements) is activated wherever spatial attention is allocated. Specifically, it is activated not only during the endogenous direction of covert attention to a cued location (e.g., the soccer ball) but also when covert attention is directed to an uncued location (e.g., the football; Van der Stigchel and Theeuwes, 2007). Spatial attention plays a major role in natural vision in facilitating the accurate targeting of saccades. It also ensures seamless perceptual transitions between discrete glances, which are accomplished by focusing resources on the saccadic goal across multiple levels of processing (Zhao et al., 2012; Bachmann and Francis, 2016). Eye movement typically precedes a motor action by a fraction of a second (Land and Hayhoe, 2001). These findings suggest that covert endogenous attention may involve attentional shifts, albeit less apparent than the shifts involved in overt attention. The differences in the outputs can thus be attributed to selectively attending to a different object or a different feature of the same object (see also Firestone and Scholl, 2016).

Taken together, the current evidence suggests that covert endogenous attention does not penetrate perceptual processes: the effects of covert attention can be attributed either to processes that resemble perceptual learning or attentional shifts. Of course, perceptual processes could be penetrated by cognitive states. The point is that the current evidence indicates that they not penetrated by covert attention.

## Author contributions

Both authors made an equal contribution to the paper.

### Conflict of interest statement

The authors declare that the research was conducted in the absence of any commercial or financial relationships that could be construed as a potential conflict of interest.

## Footnotes

^1^See, for example, Wu (in press): “If a cognitive system contains information R such that vision computes over R where this computation explains why the visual system yields O rather than some other output O_*n*_, given visual input I, then cognition cognitively penetrates vision.”

^2^This is one of the four mechanisms of perceptual learning have been identified: attention weighting, imprinting, differentiation, and unitization (Goldstone, 1998).

^3^Barbot et al. (2012) found that while the effects of exogenous attention are a function of the second-order spatial frequency content, endogenous attention affects second-order contrast sensitivity irrespective of the second-order spatial frequency content.
